# Supplementary material for: XCMAX4: A Robust X Chromosomal Genetic Association Test Accounting for Covariates
Source: Genes (Basel). 2022 May 9;13(5):847. doi: 10.3390/genes13050847 (PMC9141238; doi:10.3390/genes13050847)
Supplement: Supplementary file 1 [file genes-13-00847-s001.zip › genes-1701303supplementary materials.pdf]

Supplementary Material for "XCMAX4: A Robust X Chromosomal Genetic Association Test Accounting for Covariates"

Table S1 Estimated type Ⅰ error rate $(\times \mathbf{10^{-4}})$ at the nominal significance level 1×10^{-4} for XCMAX4, FM_{01}, FM_{02}, FM_F, and FM_S against q_f, q_m, α_2, and α_3 based on 1,000,000 replicates when $\boldsymbol{F = 0.05}$.

q_f	q_m	α_3	$\alpha_2 = 0.4005$					$\alpha_2 = -0.4005$				
			XCMAX4	FM_{01}	FM_{02}	FM_F	FM_S	XCMAX4	FM_{01}	FM_{02}	FM_F	FM_S
0.1	0.1	0.5	0.86	1.10	0.91	0.86	0.91	0.84	1.05	0.95	0.87	1.01
	0.2		0.84	0.93	0.83	0.82	0.95	0.97	1.02	1.08	1.02	0.93
	0.3		1.12	1.03	0.93	1.13	1.02	1.01	1.03	1.16	0.85	1.02
0.2	0.1		0.90	0.94	0.95	0.80	0.91	1.01	0.99	1.08	0.87	1.03
	0.2		0.82	0.93	0.94	0.91	0.90	0.96	1.00	1.05	1.05	1.04
	0.3		0.83	0.96	0.85	0.89	0.98	0.89	1.08	1.05	1.04	1.05
0.3	0.1		0.80	0.98	0.93	1.02	0.87	0.95	0.97	0.88	0.97	0.85
	0.2		0.84	0.93	0.94	1.13	1.01	1.16	1.26	1.16	1.22	1.13
	0.3		0.94	1.08	0.94	0.99	1.04	0.88	0.96	0.95	1.06	0.94
0.1	0.1	1.5	0.87	0.87	0.97	0.82	0.99	0.98	0.98	0.95	0.85	1.03
	0.2		0.97	1.13	1.03	0.98	1.03	0.84	0.88	1.04	0.81	0.91
	0.3		0.86	0.78	0.92	0.87	0.75	1.03	0.99	0.96	1.03	0.98
0.2	0.1		0.91	0.88	1.01	0.80	1.00	1.00	1.08	1.03	0.88	1.01
	0.2		0.96	0.99	0.94	0.77	1.10	0.85	0.99	1.04	0.89	0.99
	0.3		1.06	0.81	1.10	1.02	0.97	0.82	0.94	1.00	1.06	0.88
0.3	0.1		0.93	1.14	0.98	1.15	0.93	1.00	1.15	0.92	0.98	0.92
	0.2		1.07	1.21	1.07	1.14	1.13	1.03	1.33	1.07	1.02	1.18
	0.3		0.91	1.04	1.09	0.90	1.11	0.99	1.03	0.98	1.10	1.05

Table S2. Estimated type Ⅰ error rate $(\times \mathbf{10^{-3}})$ at the nominal significance level 1×10^{-3} for XCMAX4, FM_{01}, FM_{02}, FM_F, and FM_S against q_f, q_m, α_2, and α_3 based on 1,000,000 replicates under **HWE**.

q_f	q_m	α_3	$\alpha_2 = 0.4005$					$\alpha_2 = -0.4005$				
			XCMAX4	FM_{01}	FM_{02}	FM_F	FM_S	XCMAX4	FM_{01}	FM_{02}	FM_F	FM_S
0.1	0.1	0.5	0.89	0.96	0.96	0.97	0.94	0.90	1.01	0.98	0.97	0.97
	0.2		0.84	0.92	0.95	0.91	0.90	0.84	0.95	0.95	0.92	0.94
	0.3		0.92	0.97	1.00	0.99	0.97	0.97	1.12	1.09	1.03	1.10
0.2	0.1		0.95	1.01	1.02	0.97	1.02	0.83	0.92	0.88	0.90	0.90
	0.2		0.87	0.96	0.98	0.93	0.95	0.81	0.96	0.93	0.93	0.95
	0.3		0.91	1.00	1.02	0.98	1.01	0.88	0.99	1.00	0.96	0.98
0.3	0.1		0.87	0.96	0.96	0.92	0.94	0.89	1.00	0.96	0.93	0.94
	0.2		0.89	1.00	1.01	0.97	1.00	0.87	1.00	1.02	0.94	1.01
	0.3		0.85	0.94	0.95	0.96	0.95	0.96	1.03	1.03	1.09	1.03
0.1	0.1	1.5	0.90	0.99	0.95	0.92	0.97	0.85	0.97	0.93	0.92	0.95
	0.2		0.90	1.00	0.97	0.96	0.99	0.88	0.93	0.94	0.96	0.94
	0.3		0.89	0.96	1.03	1.03	0.97	0.89	1.00	0.99	0.97	1.00
0.2	0.1		0.88	0.94	0.93	0.89	0.91	0.81	0.96	0.94	0.95	0.95
	0.2		0.86	0.96	0.97	0.95	0.96	0.86	0.95	0.97	0.95	0.99
	0.3		0.88	1.05	1.02	0.98	1.03	0.92	0.99	1.04	1.01	1.02
0.3	0.1		0.91	1.00	1.01	0.97	1.00	0.89	1.04	1.01	0.91	0.99
	0.2		0.83	0.95	0.94	0.93	0.94	0.82	0.93	0.92	0.94	0.92
	0.3		0.88	1.01	0.98	1.01	0.99	0.88	1.03	1.03	1.00	1.03

Table S3. Estimated type I error rate $(\times \mathbf{10^{-5}})$ at the nominal significance level 1×10^{-5} for XCMAX4, FM_{01}, FM_{02}, FM_F, and FM_S against q_f, q_m, α_2, and α_3 based on 1,000,000 replicates under **HWE**.

q_f	q_m	α_3	$\alpha_2 = 0.4005$					$\alpha_2 = -0.4005$				
			XCMAX4	FM_{01}	FM_{02}	FM_F	FM_S	XCMAX4	FM_{01}	FM_{02}	FM_F	FM_S
0.1	0.1	0.5	0.8	0.9	1.0	0.9	1.0	0.6	0.8	0.5	0.4	0.6
	0.2		0.5	0.5	1.0	0.5	0.6	0.6	0.5	0.5	0.6	0.5
	0.3		0.9	0.7	1.0	0.4	0.8	0.5	0.3	0.8	0.4	0.2
0.2	0.1		0.8	0.8	0.6	0.6	0.6	0.6	0.8	0.8	0.5	0.8
	0.2		1.2	1.1	0.8	0.8	1.1	0.7	0.7	0.8	1.4	0.7
	0.3		0.8	0.6	1.2	0.6	0.8	0.5	0.4	0.8	0.8	0.9
0.3	0.1		0.8	0.6	0.9	0.9	0.8	1.1	1.2	1.1	1.1	1.1
	0.2		1.2	1.5	1.2	1.5	1.3	0.9	1.2	1.1	1.2	1.1
	0.3		1.1	1.0	1.0	1.0	0.9	1.0	1.2	1.1	1.1	0.8
0.1	0.1	1.5	0.4	0.4	0.8	1.1	0.6	1.1	0.8	0.8	0.7	1.0
	0.2		0.3	0.3	0.6	0.3	0.3	0.9	0.3	0.7	0.9	0.6
	0.3		0.6	1.0	0.5	0.9	1.1	1.4	1.6	1.4	1.4	1.4
0.2	0.1		0.7	0.9	1.0	0.8	0.9	1.1	1.2	1.2	0.8	1.1
	0.2		1.3	1.1	0.7	1.7	1.0	0.9	1.0	1.3	0.9	1.1
	0.3		1.0	1.2	1.0	1.3	1.4	1.2	0.9	1.1	1.4	0.8
0.3	0.1		0.7	1.5	0.9	0.4	0.8	0.8	1.2	0.8	0.6	0.6
	0.2		0.8	1.1	0.7	1.1	1.0	0.5	0.9	1.0	0.8	0.9
	0.3		0.4	0.5	0.3	0.4	0.3	0.8	0.7	0.9	1.2	0.6

Table S4. Estimated type I error rate $(\times \mathbf{10^{-3}})$ at the nominal significance level 1×10^{-3} for XCMAX4, FM_{01}, FM_{02}, FM_F, and FM_S against q_f, q_m, α_2, and α_3 based on 1,000,000 replicates when $\boldsymbol{F = 0.05}$.

q_f	q_m	α_3	$\alpha_2 = 0.4005$					$\alpha_2 = -0.4005$				
			XCMAX4	FM_{01}	FM_{02}	FM_F	FM_S	XCMAX4	FM_{01}	FM_{02}	FM_F	FM_S
0.1	0.1	0.5	0.83	0.93	0.95	0.89	0.93	0.82	0.93	0.90	0.89	0.93
	0.2		0.90	0.98	1.02	0.92	0.98	0.88	0.99	0.96	1.02	0.99
	0.3		0.91	1.02	1.04	0.99	1.01	0.87	0.97	0.96	1.01	0.97
0.2	0.1		0.90	0.97	0.98	0.96	0.97	0.96	1.02	1.09	0.99	1.07
	0.2		0.85	0.99	0.94	0.93	0.97	0.85	0.98	0.95	0.99	0.94
	0.3		0.90	0.97	0.98	1.02	0.97	0.90	1.00	0.99	0.96	1.01
0.3	0.1		0.90	0.98	0.96	0.97	0.95	0.88	1.01	1.00	0.93	0.98
	0.2		0.83	0.95	0.94	0.99	0.97	0.81	0.88	0.89	0.95	0.89
	0.3		0.91	1.05	1.06	1.02	1.04	0.80	0.95	0.90	0.94	0.93
0.1	0.1	1.5	0.89	0.95	1.00	0.95	1.00	0.84	0.91	0.93	0.89	0.93
	0.2		0.93	1.03	1.00	0.98	1.04	0.90	1.00	0.97	0.97	0.99
	0.3		0.84	0.94	0.93	0.94	0.93	0.88	1.00	1.01	1.04	1.00
0.2	0.1		0.91	0.99	1.04	0.91	1.02	0.89	1.02	1.00	0.92	0.99
	0.2		0.83	1.00	0.94	0.92	0.94	0.86	0.94	0.99	0.95	0.97
	0.3		0.90	1.01	0.97	1.00	1.01	0.87	0.97	0.99	0.96	0.95
0.3	0.1		0.87	0.94	0.94	0.92	0.93	0.90	0.98	1.04	0.98	1.03
	0.2		0.91	1.05	1.01	1.04	1.00	0.90	1.01	1.00	0.97	0.98
	0.3		0.87	1.02	0.98	0.98	1.01	0.87	1.02	0.98	1.00	0.97

Table S5. Estimated type I error rate $(\times \mathbf{10^{-5}})$ at the nominal significance level 1×10^{-5} for XCMAX4, FM_{01}, FM_{02}, FM_F, and FM_S against q_f, q_m, α_2, and α_3 based on 1,000,000 replicates when $\boldsymbol{F} = \mathbf{0.05}$.

q_f	q_m	α_3	$\alpha_2 = 0.4005$					$\alpha_2 = -0.4005$				
			XCMAX4	FM_{01}	FM_{02}	FM_F	FM_S	XCMAX4	FM_{01}	FM_{02}	FM_F	FM_S
0.1	0.1	0.5	1.0	0.8	1.4	0.8	1.1	1.2	0.4	0.8	0.5	0.5
	0.2		0.8	0.7	0.8	1.2	0.7	0.8	0.7	1.0	1.0	0.9
	0.3		0.6	1.1	0.9	1.0	1.1	1.4	1.1	1.2	0.6	1.1
0.2	0.1		1.1	1.5	0.9	1.0	0.9	0.9	0.8	1.1	0.4	0.9
	0.2		2.0	1.1	0.6	0.9	1.1	1.0	1.0	1.3	1.5	1.0
	0.3		0.4	0.5	0.7	0.8	0.6	0.7	0.5	0.9	0.4	1.0
0.3	0.1		0.8	1.1	0.7	0.3	0.7	0.8	0.8	1.2	0.6	1.3
	0.2		0.8	0.8	0.7	1.2	0.8	1.7	2.2	2.2	2.1	1.8
	0.3		1.3	1.1	1.1	1.8	1.4	0.8	1.5	1.2	1.2	1.1
0.1	0.1	1.5	1.0	0.9	0.7	1.4	0.7	1.0	1.0	0.7	0.7	0.9
	0.2		1.4	1.3	1.0	1.1	1.1	1.0	1.1	1.0	0.3	1.0
	0.3		0.6	0.6	0.5	0.7	0.6	0.9	1.3	0.8	1.2	1.3
0.2	0.1		1.0	0.6	0.7	1.1	0.7	1.3	0.7	1.1	1.1	1.1
	0.2		0.7	1.2	1.1	0.7	1.0	1.2	1.4	1.2	0.9	1.4
	0.3		1.5	1.0	1.1	1.1	0.8	0.8	0.8	1.0	1.1	1.2
0.3	0.1		1.2	1.1	1.1	0.9	0.8	0.8	1.5	1.0	0.9	1.2
	0.2		1.3	1.4	1.2	1.5	1.2	2.0	1.9	1.7	1.2	1.9
	0.3		0.8	0.9	1.0	1.1	0.9	1.5	1.3	0.9	1.7	1.2

Table S6 Time used to test 2000 SNPs with a sample size of 5000*

**Time** (second)	XCMAX4	FM_{01}	FM_{02}	FM_F	FM_S
User	9.79	19.39	19.28	19.36	19.35
System	0.00	0.00	0.00	0.02	0.02
Elapsed	9.79	19.29	19.29	19.38	19.36

* The fitted model is the same as that in our simulation study. The results are obtained in R 4.1.2, running on a laptop with CPU: AMD Ryzen 5 5600H and RAM: 16G.

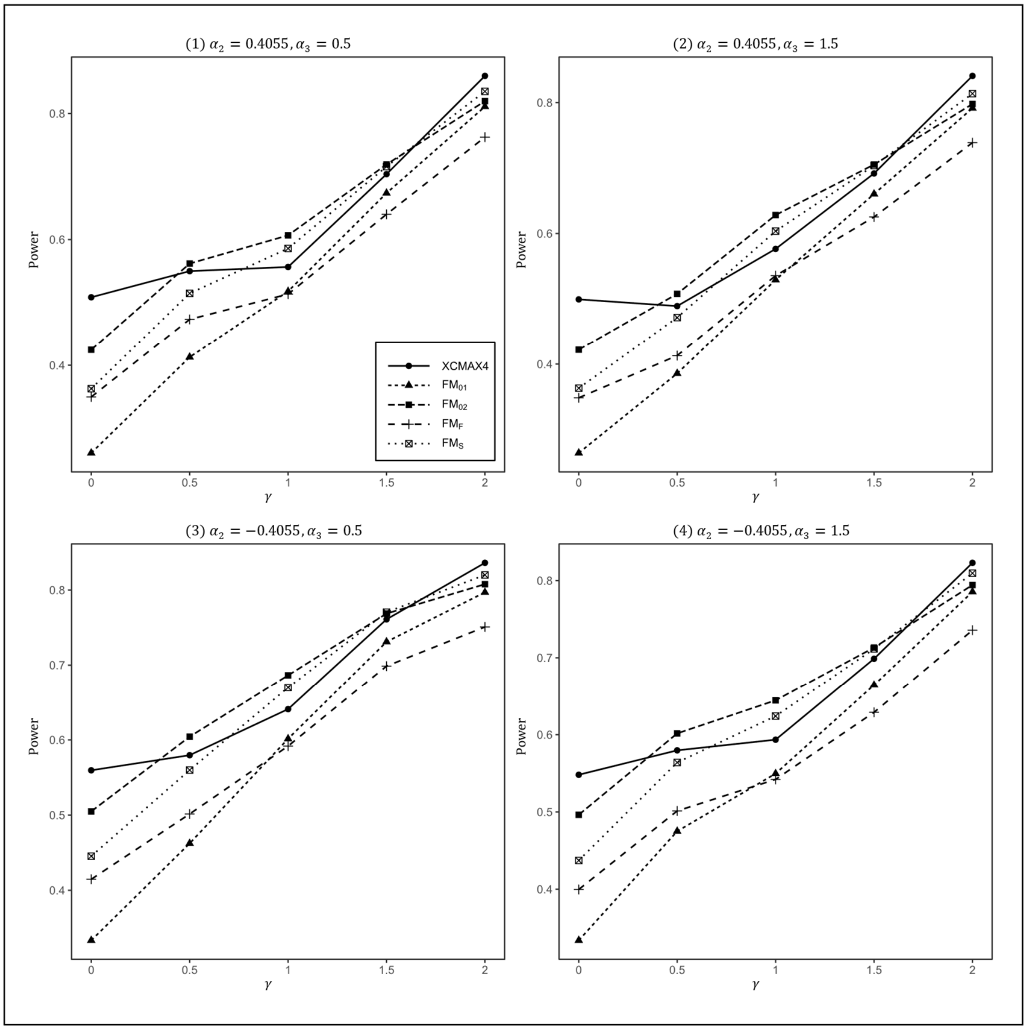

Figure S1. Estimated powers of XCMAX4, FM_{01}, FM_{02}, FM_F, and FM_S under various XCI models**.** The simulation is based on 10,000 replicates with $\boldsymbol{\beta} = \mathbf{0.15}$, $\alpha_1 = -5$, $\boldsymbol{F} = \mathbf{0.05}$, and $q_f = q_m = 0.3$.

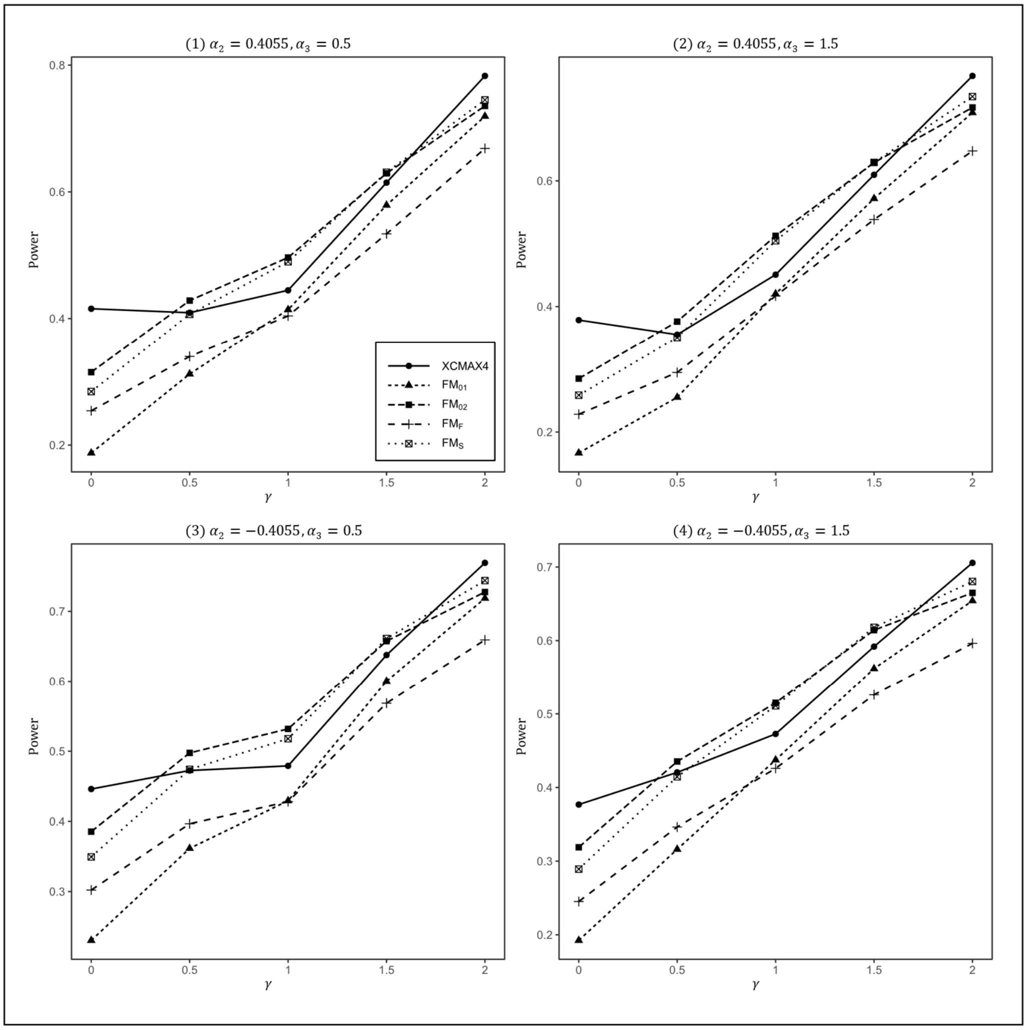

Figure S2. Estimated powers of XCMAX4, FM_{01}, FM_{02}, FM_F, and FM_S under various XCI models**.** The simulation is based on 10,000 replicates with $\boldsymbol{\beta} = \mathbf{0.15}$, $\alpha_1 = -5$, $\boldsymbol{F} = \mathbf{0.05}$, $q_f = 0.3$, and $q_m = 0.2$.

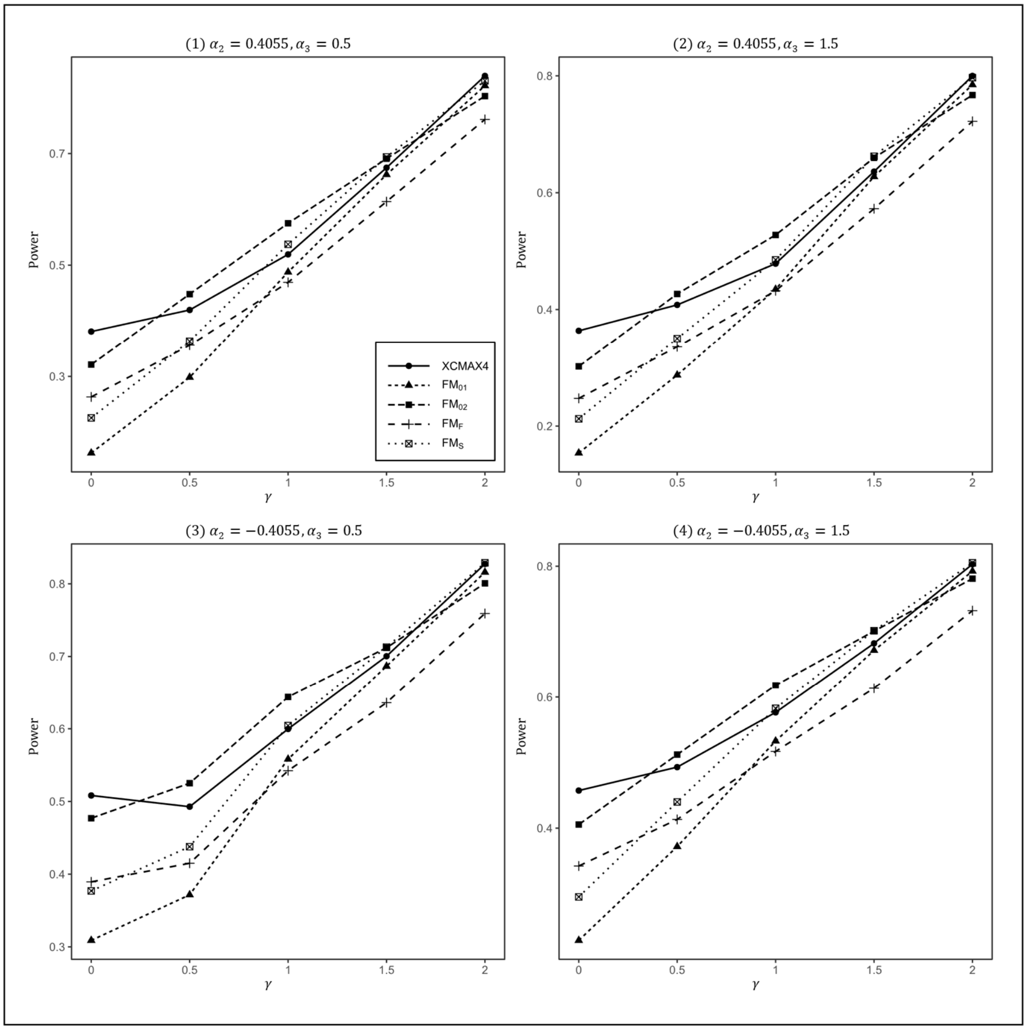

Figure S3. Estimated powers of XCMAX4, FM_{01}, FM_{02}, FM_F, and FM_S under various XCI models**.** The simulation is based on 10,000 replicates with $\boldsymbol{\beta} = \mathbf{0.15}$, $\alpha_1 = -5$, $\boldsymbol{F} = \mathbf{0.05}$, $q_f = 0.2$, and $q_m = 0.3$.

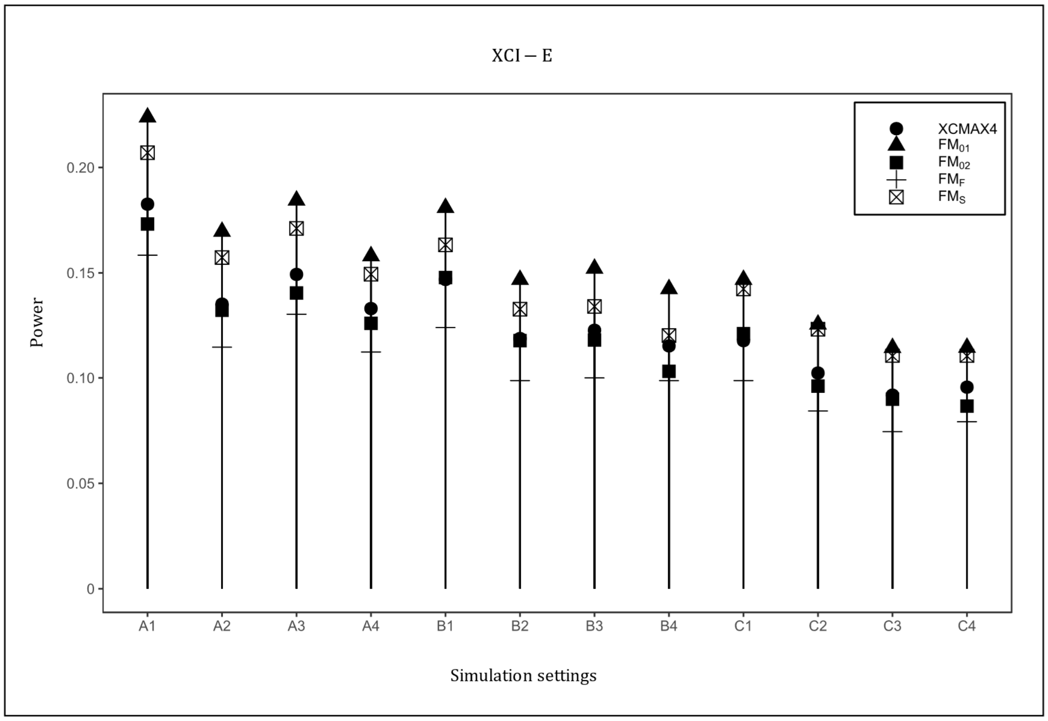

Figure S4. Powers of XCMAX4, FM_{01}, FM_{02}, FM_F, and FM_S under the XCI-E pattern. The simulation is based on 10,000 replicates with $\boldsymbol{\beta = 0.15}$, $\alpha_1 = -5$, and $\boldsymbol{F = 0.05}$. In the horizontal coordinates, "A", "B" and "C" represent three combinations of (q_f, q_m): (0.3,0.3), (0.3,0.2), and (0.2,0.3), respectively. The numbers 1-4 represent four combinations of (α_2, α_3): (0.4055,0.5), (0.4055,1.5), $(-0.4055, 0.5)$, and $(-0.4055,1.5)$, respectively.

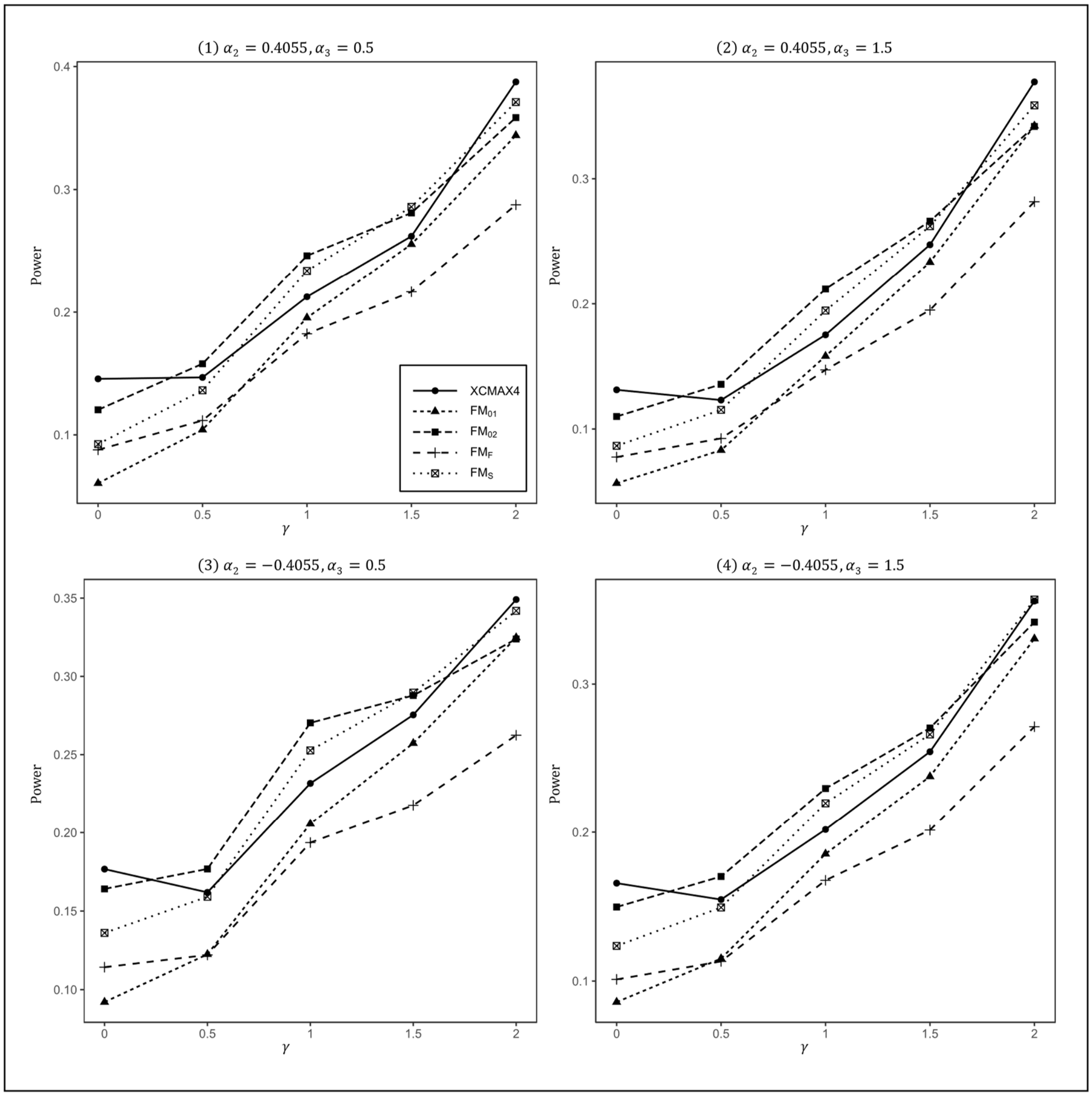

Figure S5. Estimated powers of XCMAX4, FM_{01}, FM_{02}, FM_F, and FM_S under various XCI models. The simulation is based on 10,000 replicates with $\boldsymbol{\beta} = \mathbf{0.1116}$, $\alpha_1 = -5$, $\boldsymbol{F} = \mathbf{0}$, and $q_f = q_m = 0.3$.

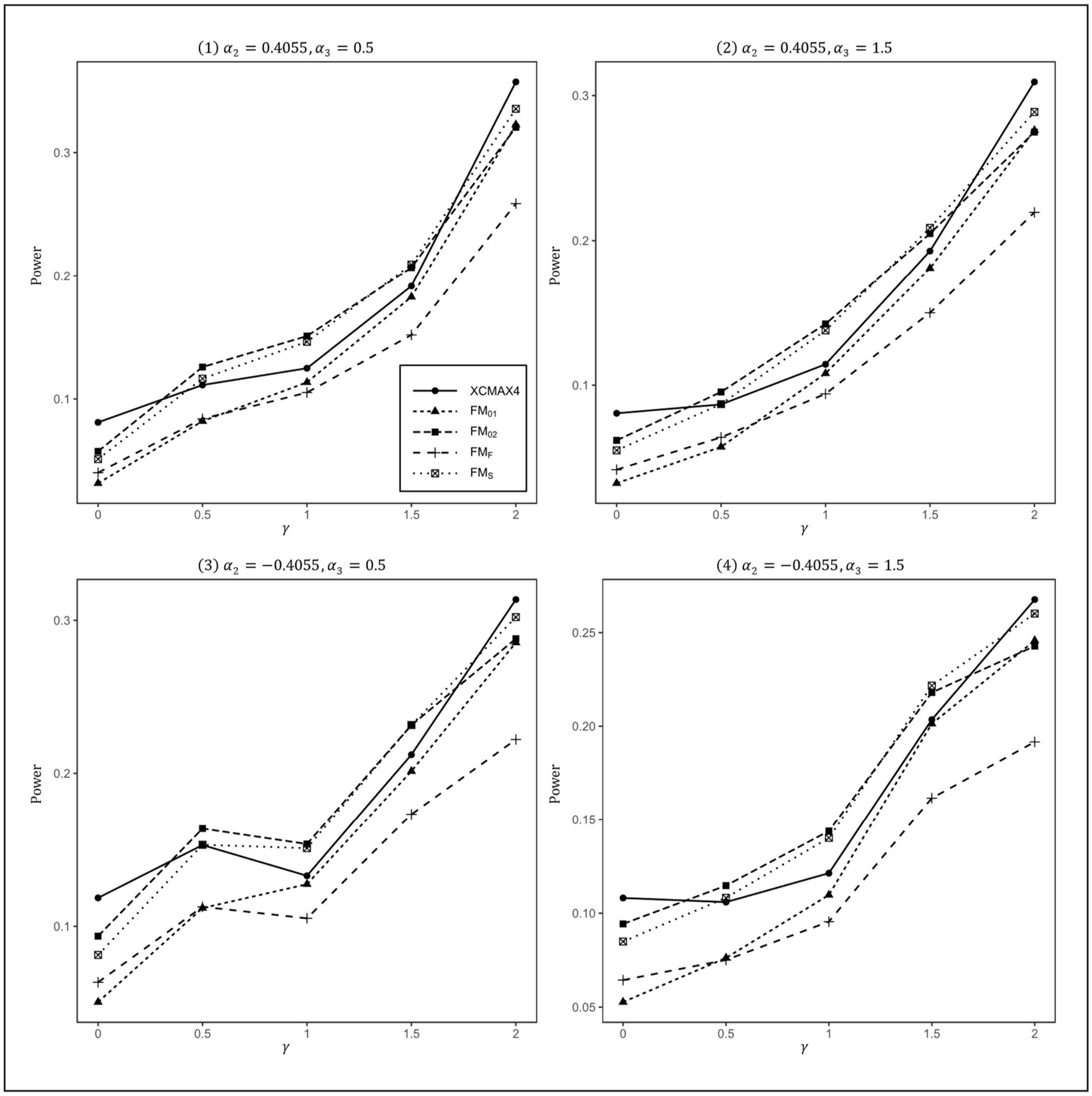

Figure S6. Estimated powers of XCMAX4, FM_{01}, FM_{02}, FM_F, and FM_S under various XCI models. The simulation is based on 10,000 replicates with $\boldsymbol{\beta} = \mathbf{0.1116}$, $\alpha_1 = -5$, $\boldsymbol{F} = \mathbf{0}$, $q_f = 0.3$, and $q_m = 0.2$.

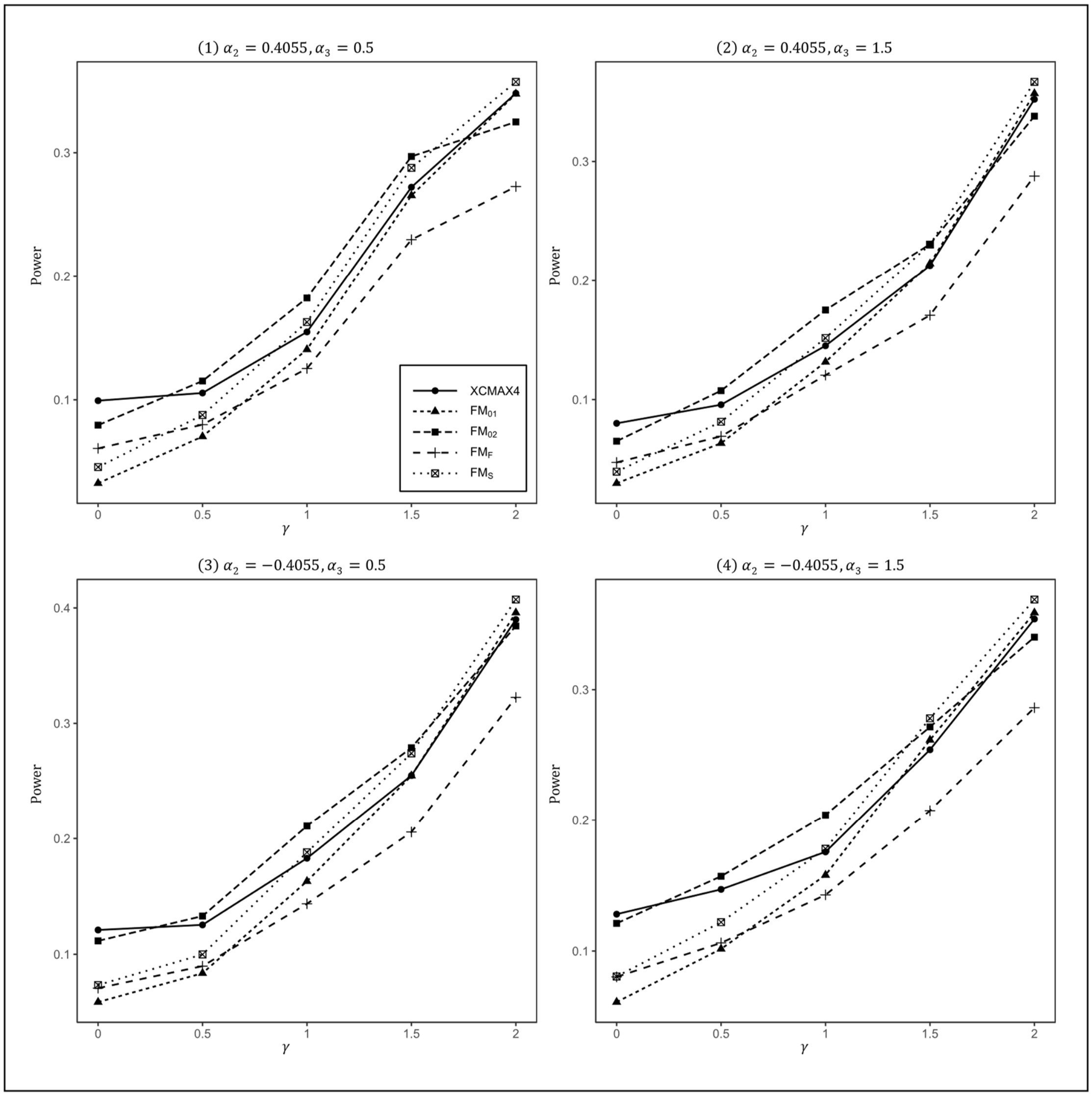

Figure S7. Estimated powers of XCMAX4, FM_{01}, FM_{02}, FM_F, and FM_S under various XCI models. The simulation is based on 10,000 replicates with $\boldsymbol{\beta} = \mathbf{0.1116}$, $\alpha_1 = -5$, $\boldsymbol{F} = \mathbf{0}$, $q_f = 0.2$, and $q_m = 0.3$.

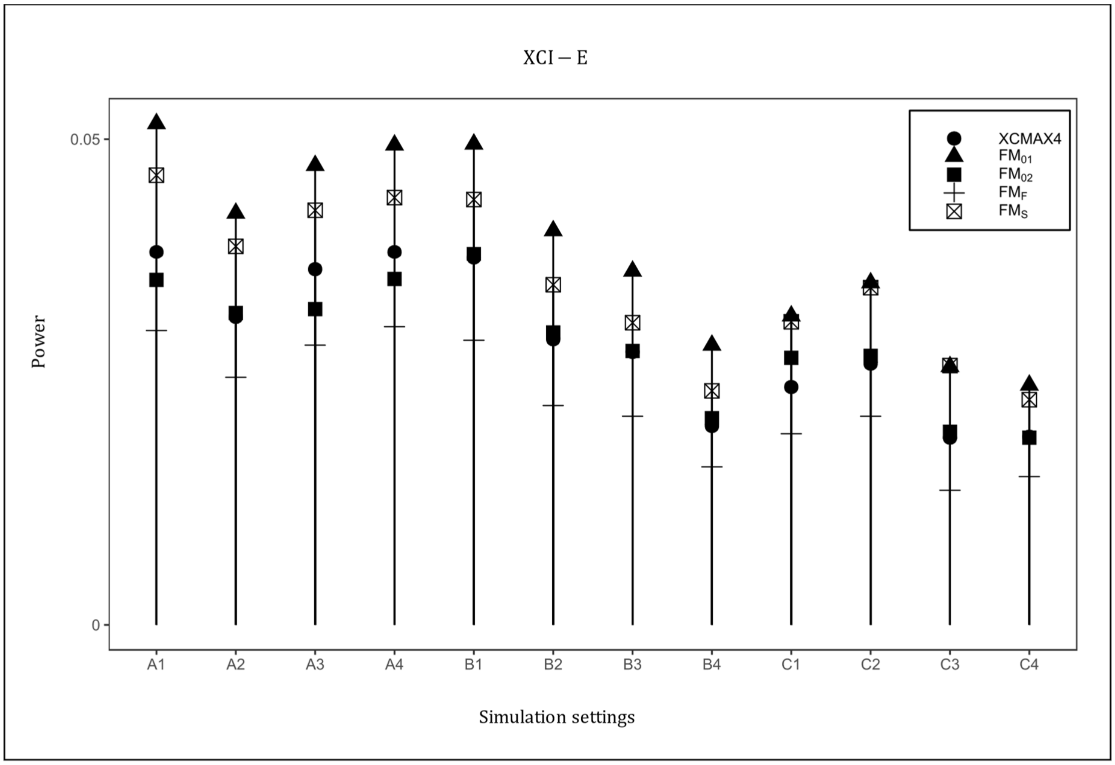

Figure S8. Powers of XCMAX4, FM_{01}, FM_{02}, FM_F, and FM_S under XCI-E. The simulation is based on 10,000 replicates with $\boldsymbol{\beta = 0.1116}$, $\alpha_1 = -5$, and $\boldsymbol{F = 0}$. In the horizontal coordinates, "A", "B" and "C" represent three combinations of (q_f, q_m): (0.3,0.3), (0.3,0.2), and (0.2,0.3), respectively, and the numbers 1-4 represent four combinations of (α_2, α_3): (0.4055,0.5), (0.4055,1.5), (−0.4055, 0.5), and (−0.4055,1.5), respectively.

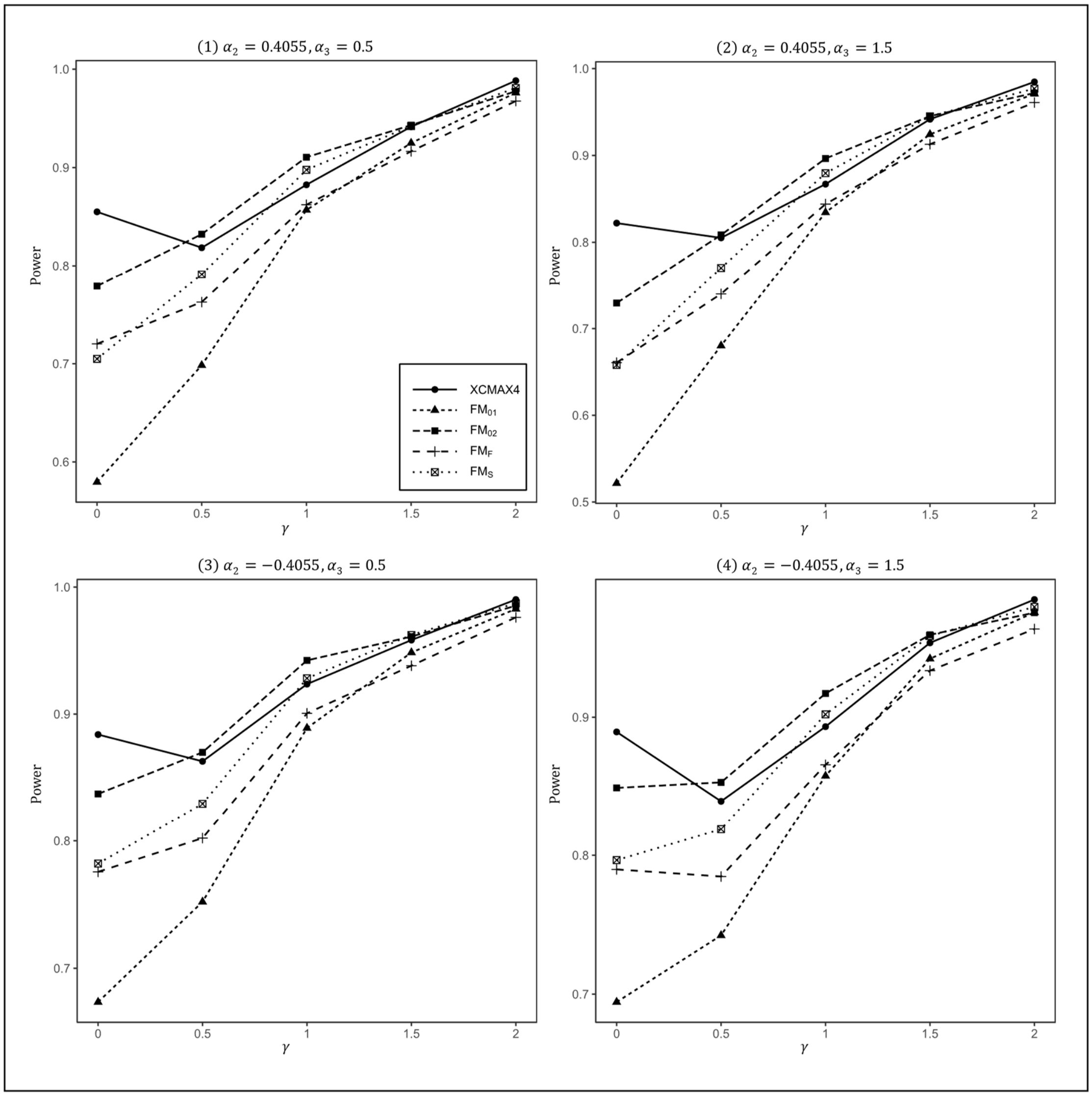

Figure S9. Estimated powers of XCMAX4, FM_{01}, FM_{02}, FM_F, and FM_S under various XCI models. The simulation is based on 10,000 replicates with $\boldsymbol{\beta} = \mathbf{0.1858}$, $\alpha_1 = -5$, $\boldsymbol{F} = \mathbf{0}$, and $q_f = q_m = 0.3$.

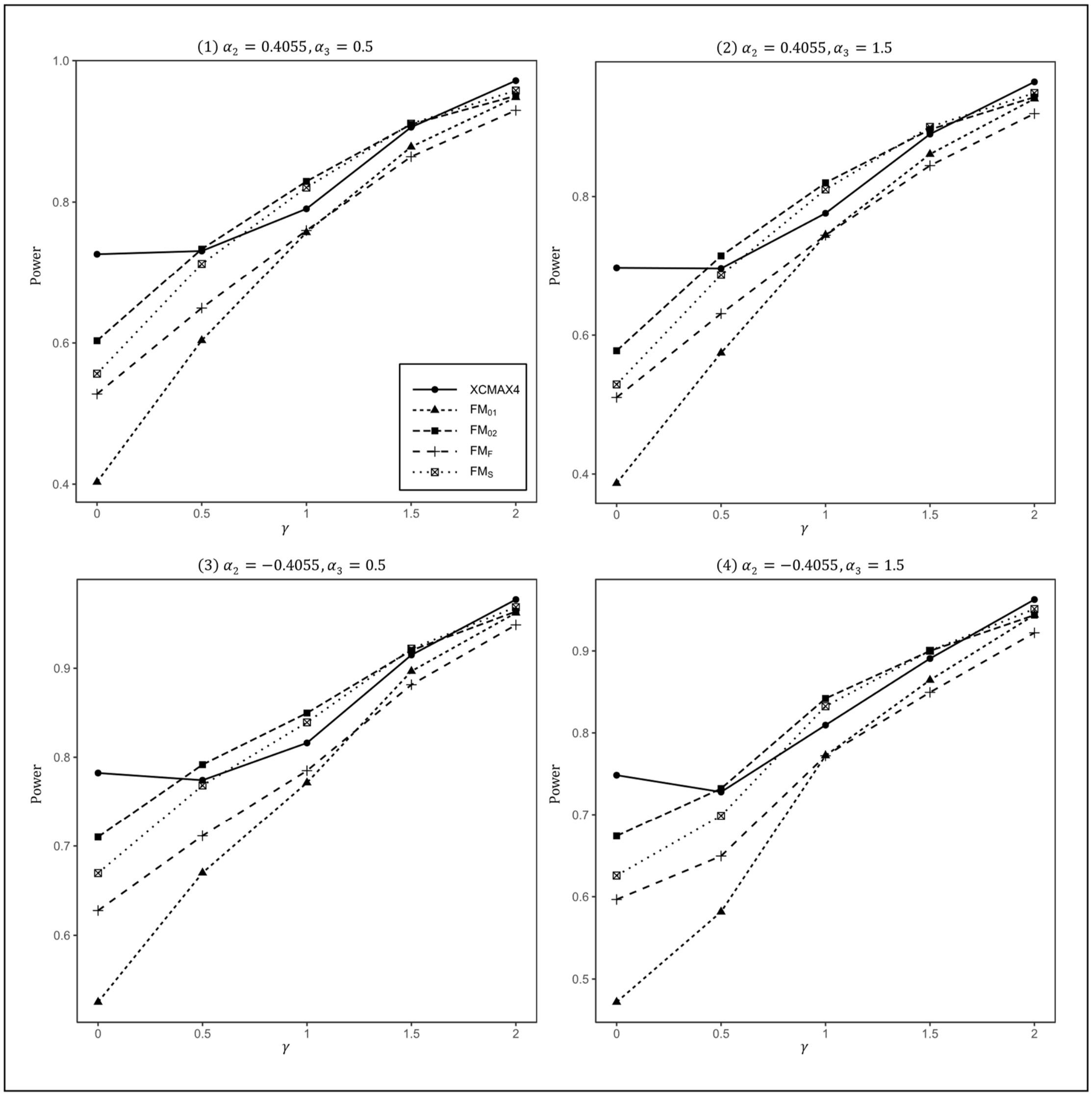

Figure S10. Estimated powers of XCMAX4, FM_{01}, FM_{02}, FM_F, and FM_S under various XCI models. The simulation is based on 10,000 replicates with $\boldsymbol{\beta} = \mathbf{0.1858}$, $\alpha_1 = -5$, $\boldsymbol{F} = \mathbf{0}$, $q_f = 0.3$, and $q_m = 0.2$.

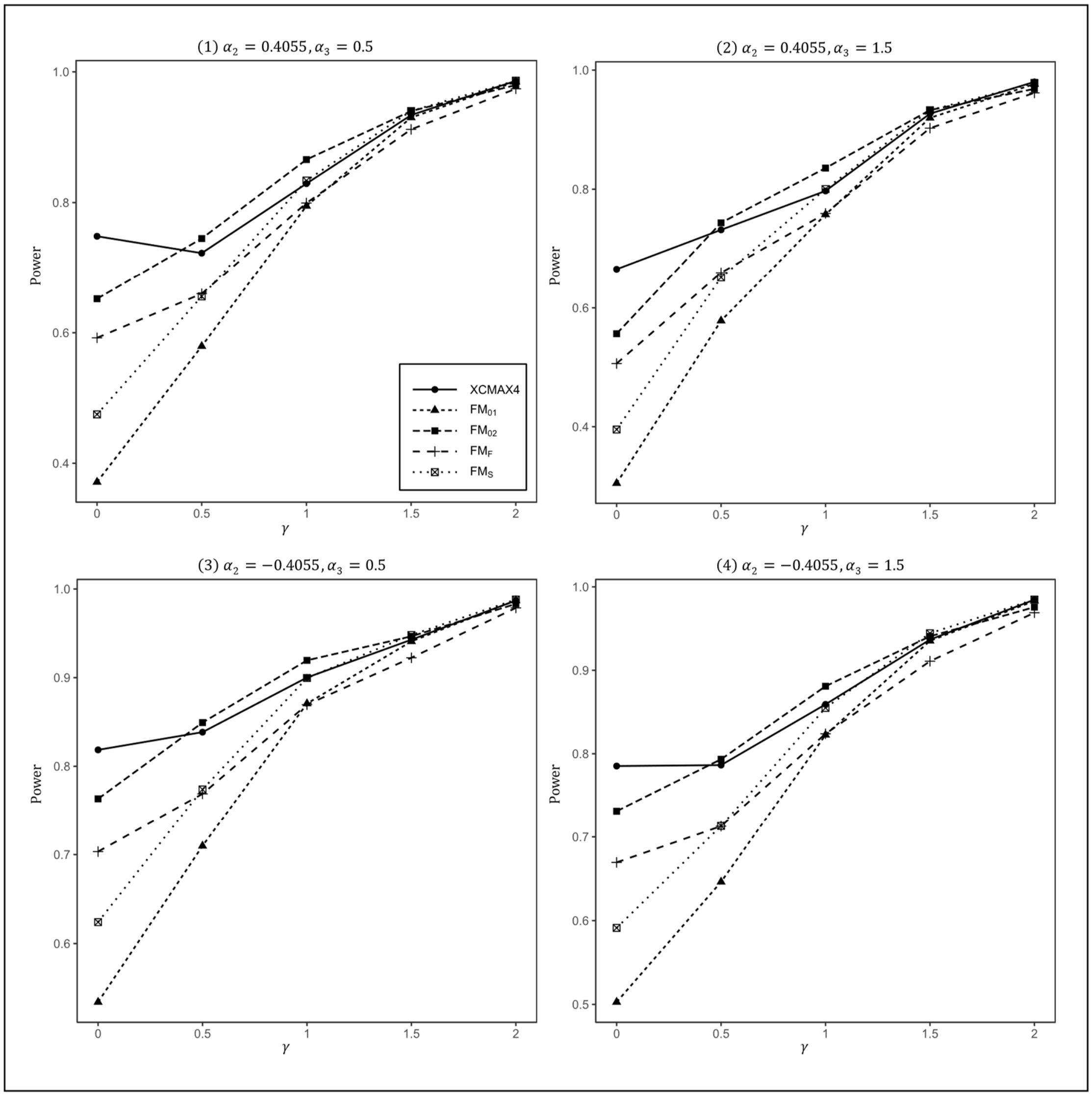

Figure S11. Estimated powers of XCMAX4, FM_{01}, FM_{02}, FM_F, and FM_S under various XCI models. The simulation is based on 10,000 replicates with $\boldsymbol{\beta} = \mathbf{0.1858}$, $\alpha_1 = -5$, $\boldsymbol{F} = \mathbf{0}$, $q_f = 0.2$, and $q_m = 0.3$.

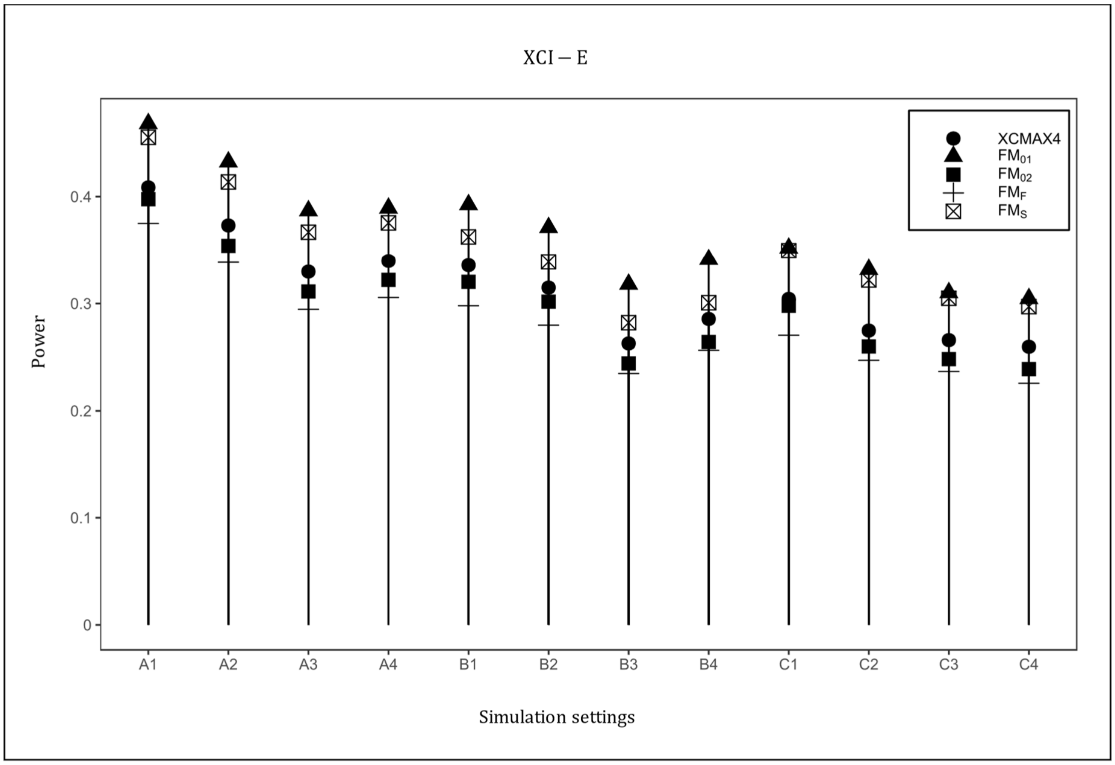

Figure S12. Powers of XCMAX4, FM_{01}, FM_{02}, FM_{F}, and FM_{S} under XCI-E. The simulation is based on 10,000 replicates with $\boldsymbol{\beta} = \mathbf{0.1858}$, $\alpha_1 = -5$, and $\boldsymbol{F} = \mathbf{0}$. In the horizontal coordinates, "A", "B" and "C" represent three combinations of (q_f, q_m): (0.3,0.3), (0.3,0.2), and (0.2,0.3), respectively, and the numbers 1-4 represent four combinations of (α_2, α_3): (0.4055,0.5), (0.4055,1.5), (−0.4055, 0.5), and (−0.4055,1.5), respectively.

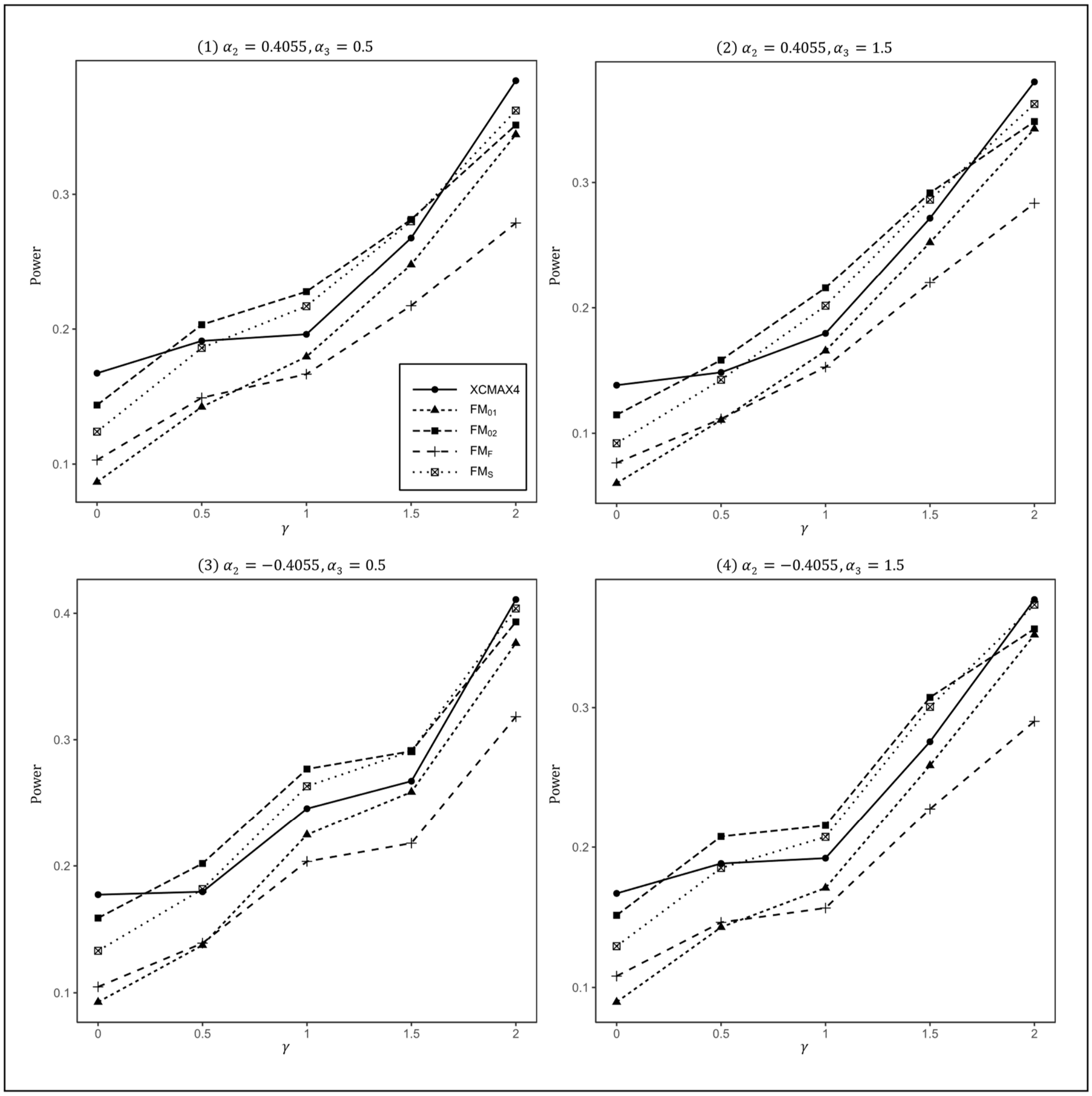

Figure S13. Estimated powers of XCMAX4, FM_{01}, FM_{02}, FM_F, and FM_S under various XCI models**.** The simulation is based on 10,000 replicates with $\boldsymbol{\beta} = \mathbf{0.1116}$, $\alpha_1 = -5$, $\boldsymbol{F} = \mathbf{0.05}$, and $q_f = q_m = 0.3$.

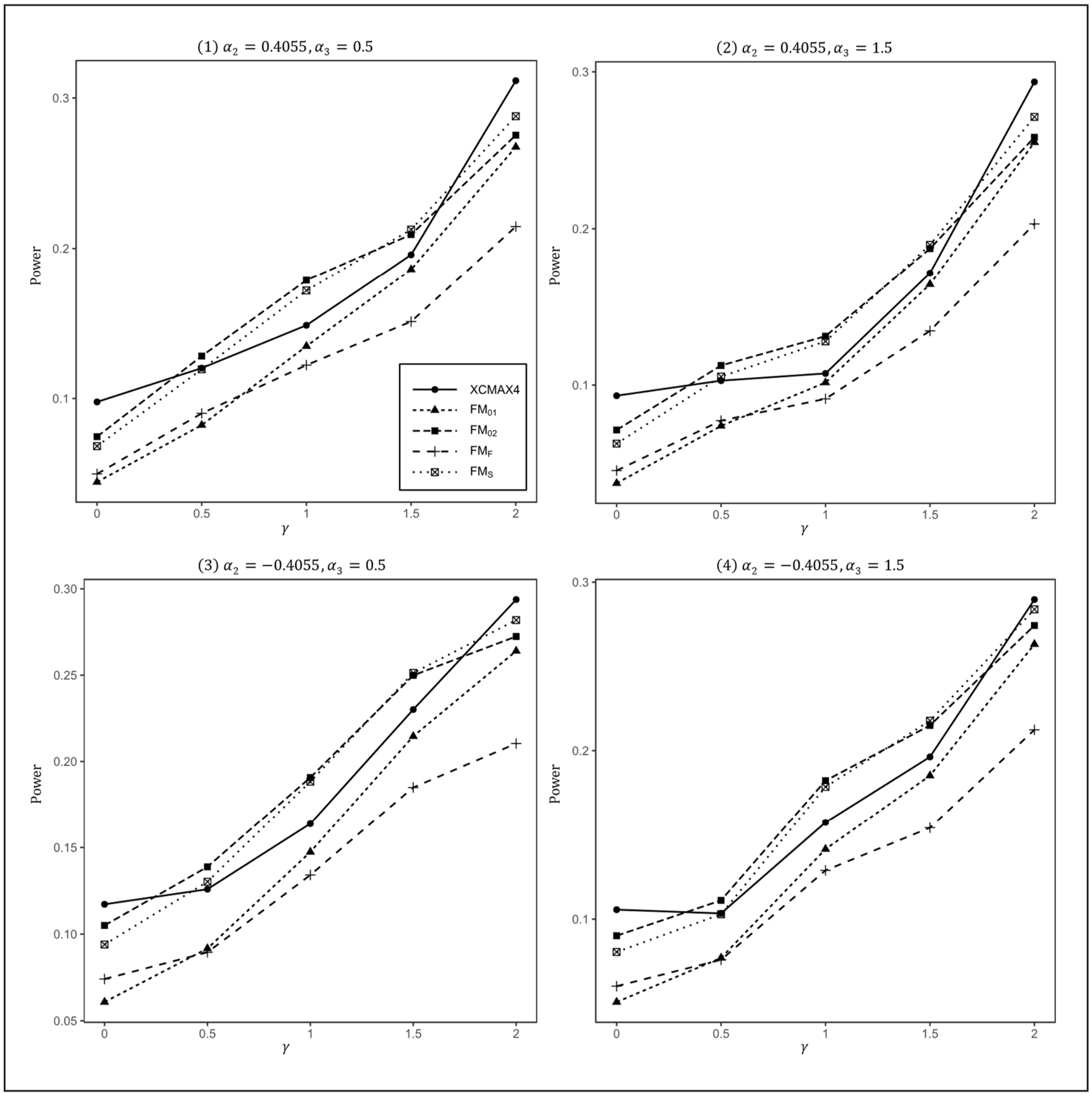

Figure S14. Estimated powers of XCMAX4, FM_{01}, FM_{02}, FM_F, and FM_S under various XCI models. The simulation is based on 10,000 replicates with $\boldsymbol{\beta} = \mathbf{0.1116}$, $\alpha_1 = -5$, $\boldsymbol{F} = \mathbf{0.05}$, $q_f = 0.3$, and $q_m = 0.2$.

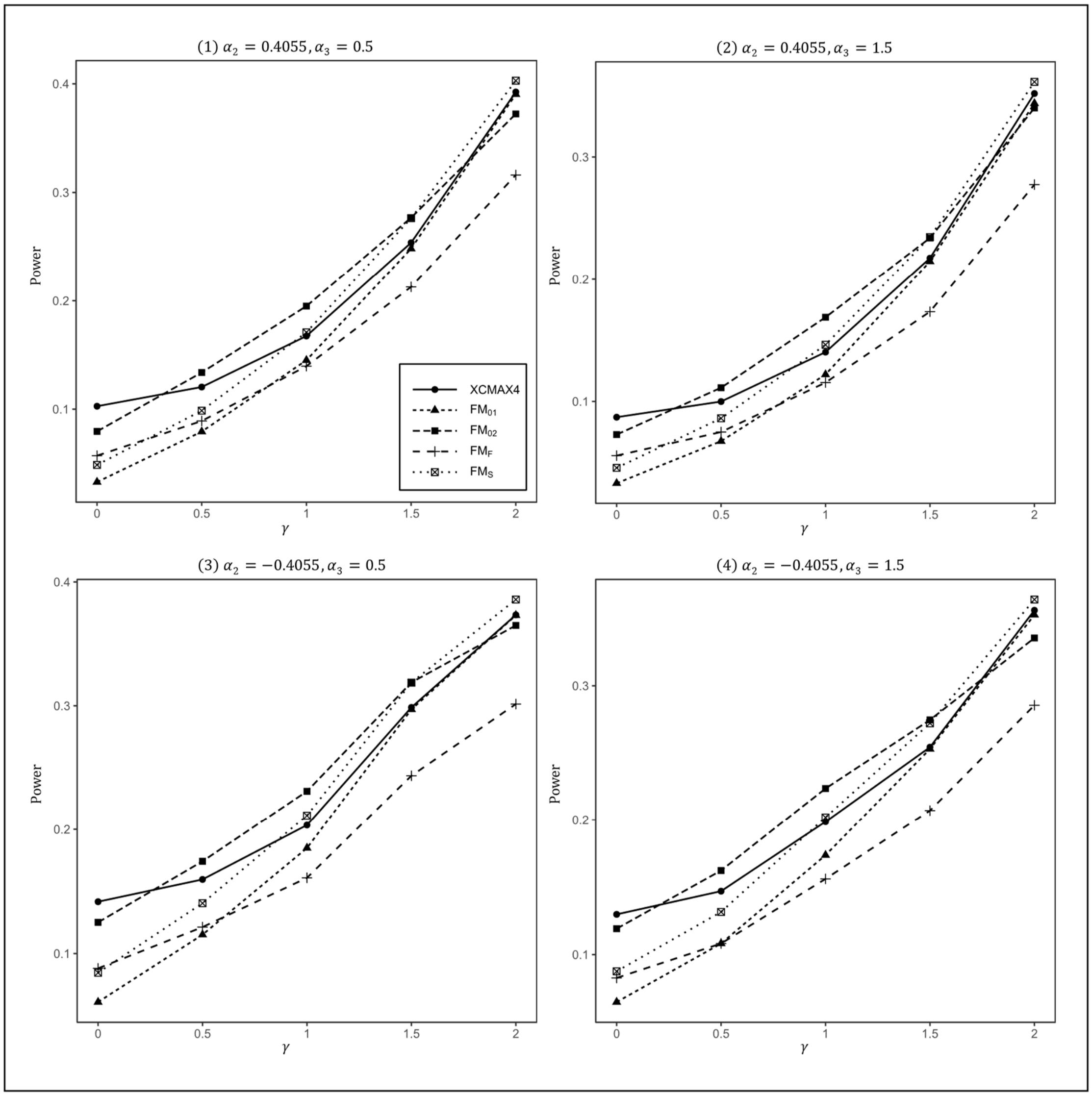

Figure S15. Estimated powers of XCMAX4, FM_{01}, FM_{02}, FM_F, and FM_S under various XCI models. The simulation is based on 10,000 replicates with $\boldsymbol{\beta} = \mathbf{0.1116}$, $\alpha_1 = -5$, $\boldsymbol{F} = \mathbf{0.05}$, $q_f = 0.2$, and $q_m = 0.3$.

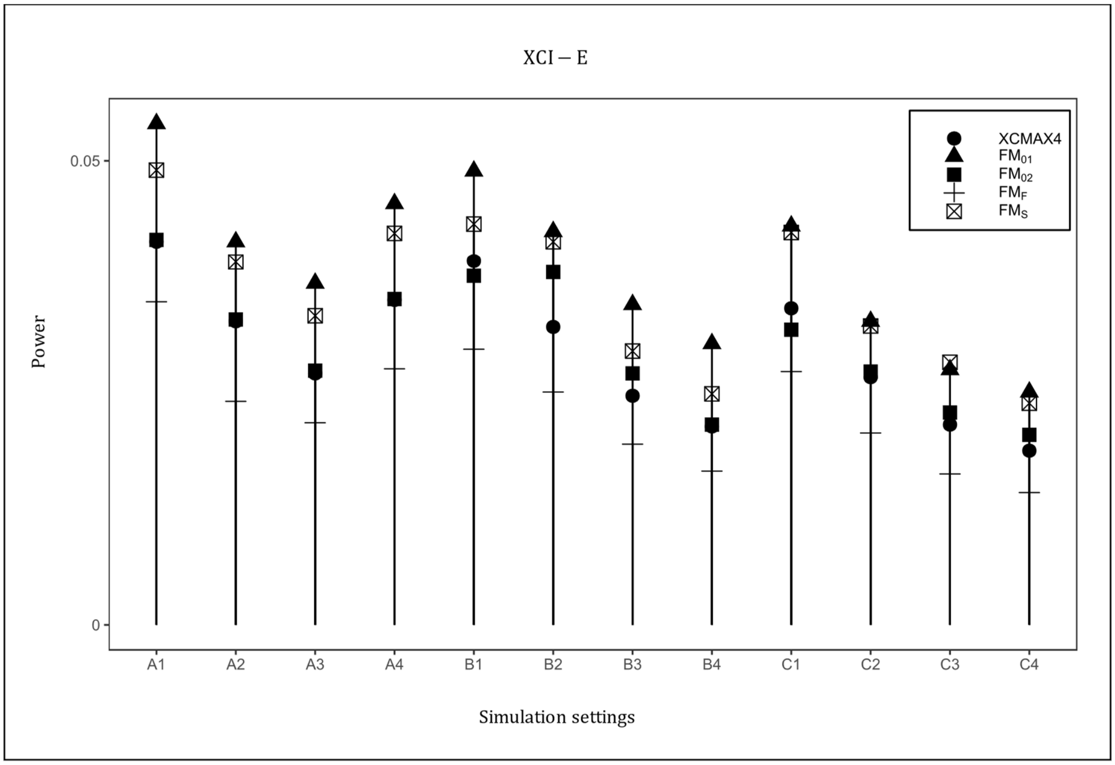

Figure S16. Powers of XCMAX4, FM_{01}, FM_{02}, FM_F, and FM_S under XCI-E. The simulation is based on 10,000 replicates with $\boldsymbol{\beta} = \mathbf{0.1116}$, $\alpha_1 = -5$, and $\boldsymbol{F} = \mathbf{0.05}$. In the horizontal coordinates, "A", "B" and "C" represent three combinations of (q_f, q_m): (0.3,0.3), (0.3,0.2), and (0.2,0.3), respectively, and the numbers 1-4 represent four combinations of (α_2, α_3): (0.4055,0.5), (0.4055,1.5), $(-0.4055, 0.5)$, and $(-0.4055,1.5)$, respectively.

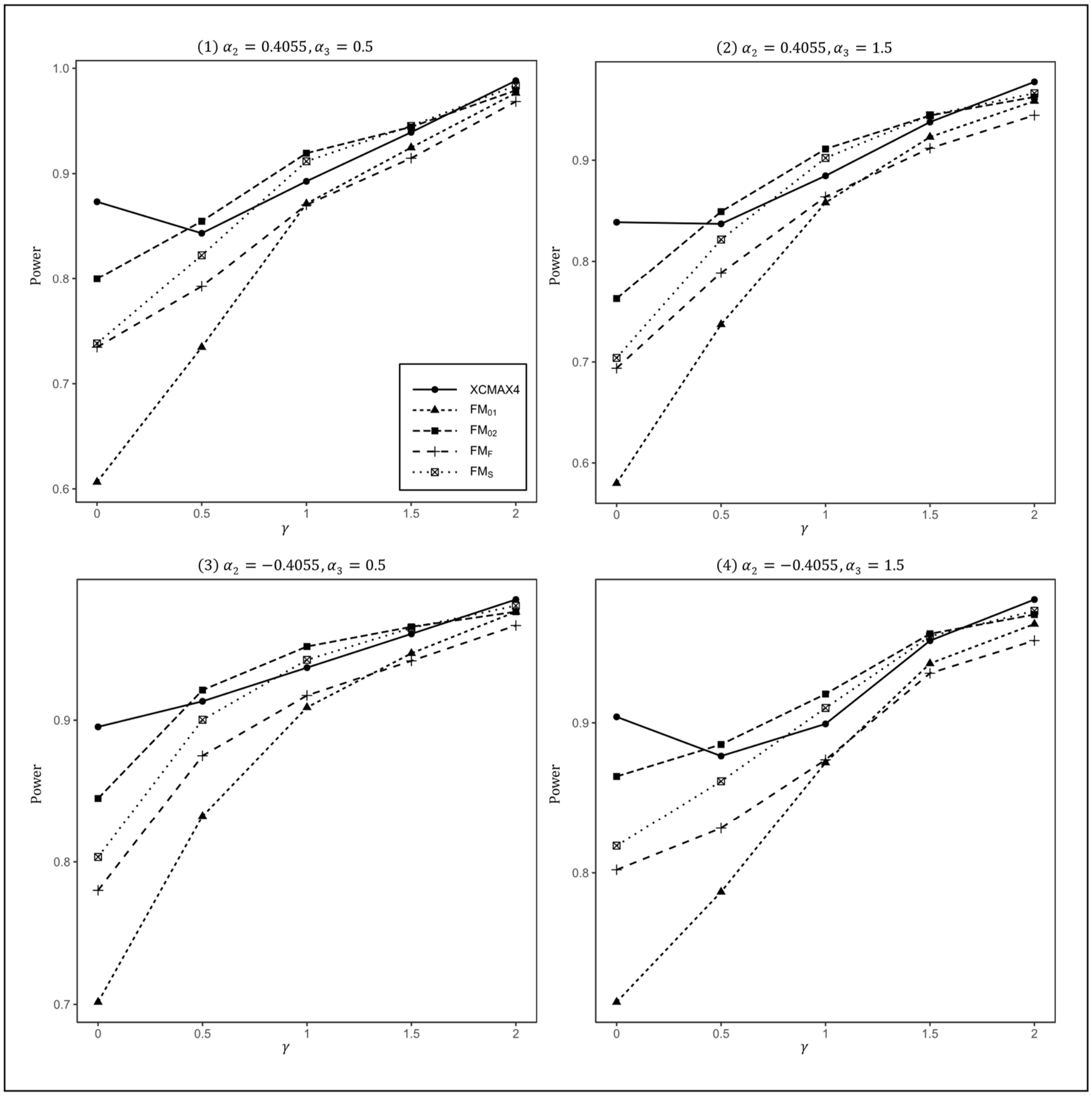

Figure S17. Estimated powers of XCMAX4, FM_{01}, FM_{02}, FM_F, and FM_S under various XCI models**.** The simulation is based on 10,000 replicates with $\boldsymbol{\beta} = \mathbf{0.1858}$, $\alpha_1 = -5$, $\boldsymbol{F} = \mathbf{0.05}$, and $q_f = q_m = 0.3$.

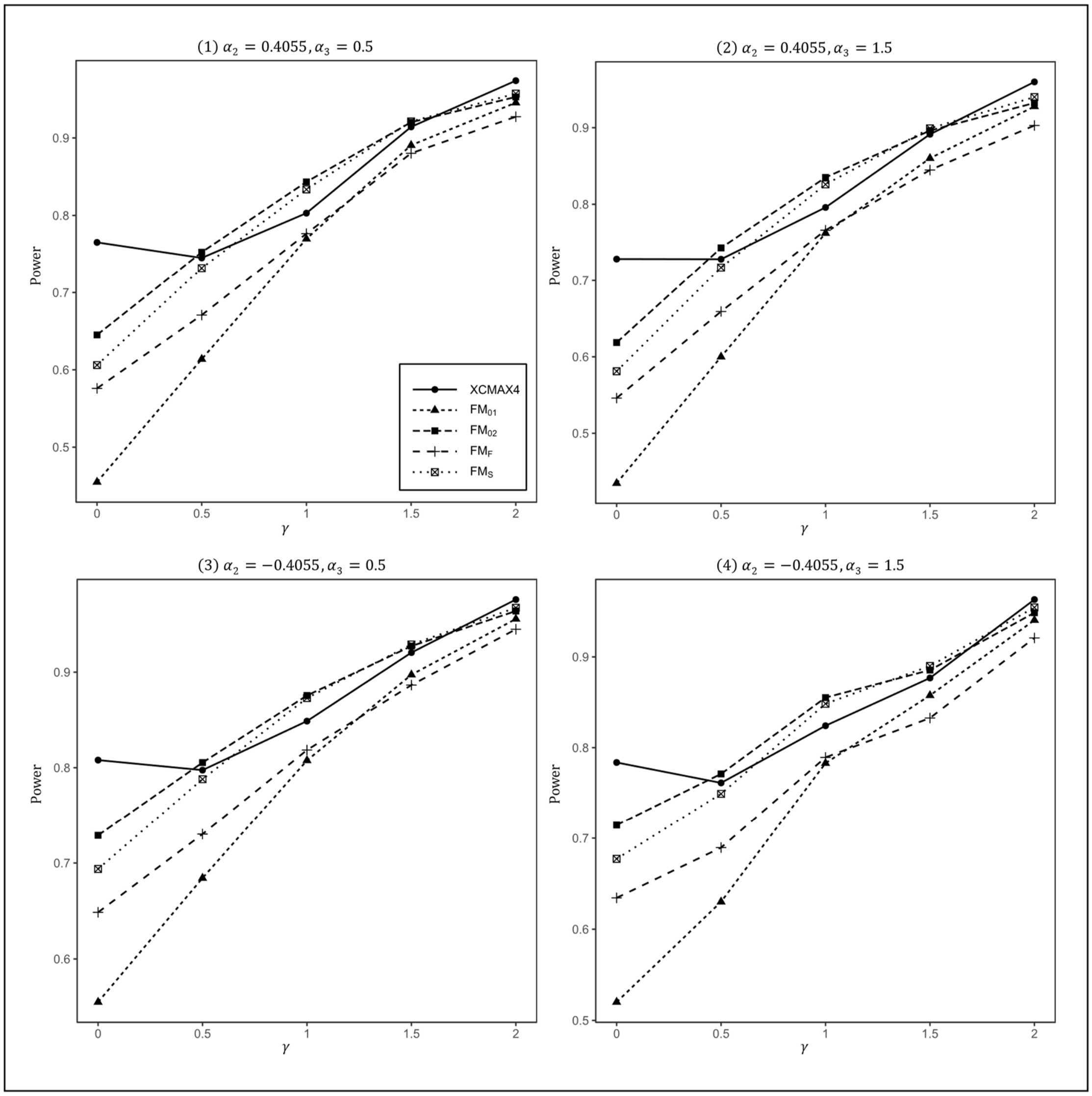

Figure S18. Estimated powers of XCMAX4, FM_{01}, FM_{02}, FM_F, and FM_S under various XCI models. The simulation is based on 10,000 replicates with $\boldsymbol{\beta} = \mathbf{0.1858}$, $\alpha_1 = -5$, $\boldsymbol{F} = \mathbf{0.05}$, $q_f = 0.3$, and $q_m = 0.2$.

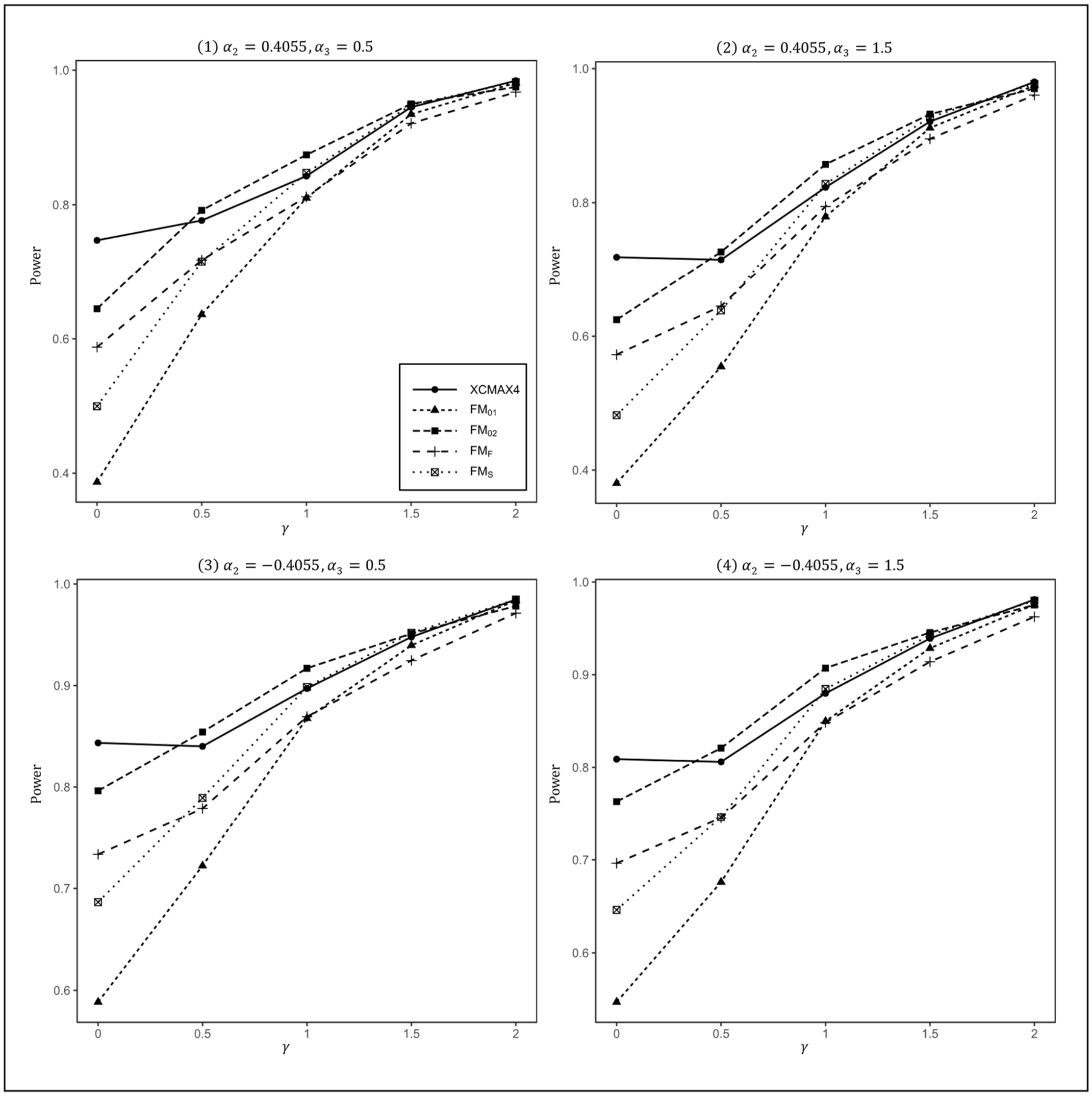

Figure S19. Estimated powers of XCMAX4, FM_{01}, FM_{02}, FM_F, and FM_S under various XCI models. The simulation is based on 10,000 replicates with $\boldsymbol{\beta} = \mathbf{0.1858}$, $\alpha_1 = -5$, $\boldsymbol{F} = \mathbf{0.05}$, $q_f = 0.2$, and $q_m = 0.3$.

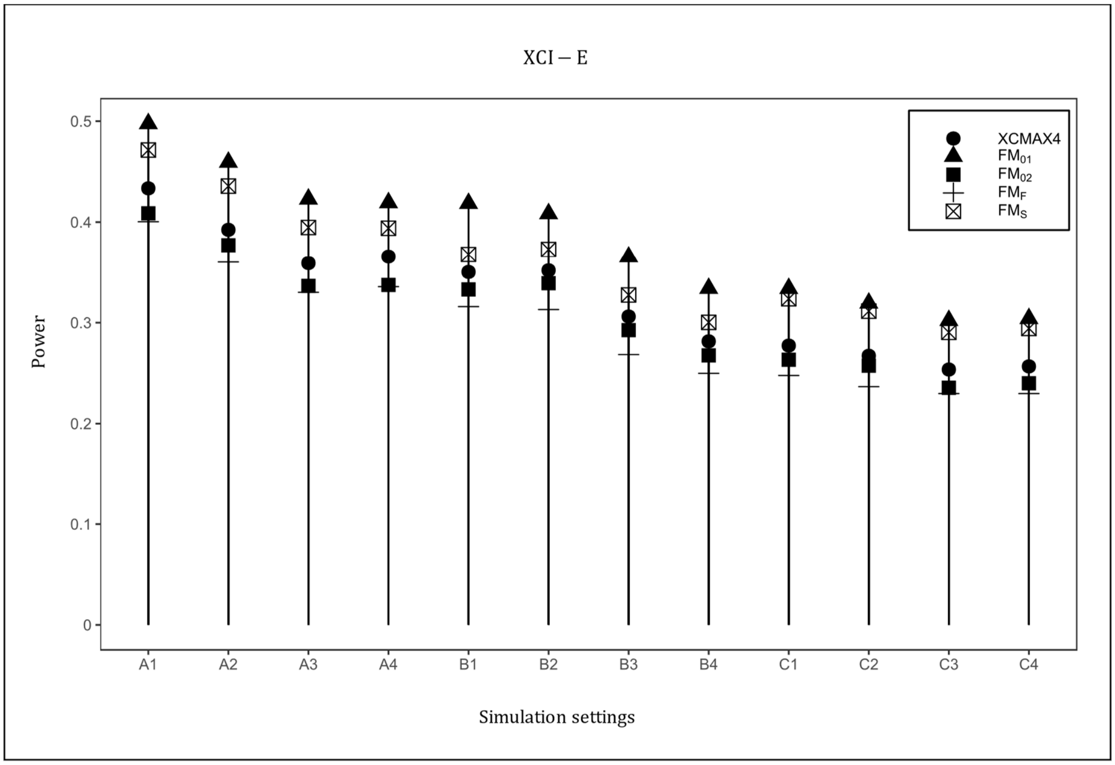

Figure S20. Powers of XCMAX4, FM_{01}, FM_{02}, FM_F, and FM_S under XCI-E. The simulation is based on 10,000 replicates with $\boldsymbol{\beta = 0.1858}$, $\alpha_1 = -5$, and $\boldsymbol{F = 0.05}$. In the horizontal coordinates, "A", "B" and "C" represent three combinations of (q_f, q_m): (0.3,0.3), (0.3,0.2), and (0.2,0.3), respectively, and the numbers 1-4 represent four combinations of (α_2, α_3): (0.4055,0.5), (0.4055,1.5), (−0.4055, 0.5), and (−0.4055,1.5), respectively.
